# Challenges and Opportunities With the Use of Hypofractionated Radiation Therapy in Cancer Care: Regional Perspectives From South Korea, Japan, Singapore, and Australia

**DOI:** 10.1016/j.adro.2023.101291

**Published:** 2023-06-14

**Authors:** Tetsuo Akimoto, Hidefumi Aoyama, Melvin L.K. Chua, Dasantha Jayamanne, Takashi Mizowaki, Lucinda Morris, Hiroshi Onishi, Si Yeol Song, Youssef H. Zeidan, Ricky A. Sharma

**Affiliations:** a Division of Radiation Oncology and Particle Therapy, National Cancer Center Hospital East, Chiba, Japan; b Department of Radiation Oncology, Faculty of Medicine and Graduate School of Medicine, Hokkaido University, Sapporo, Hokkaido, Japan; c Division of Radiation Oncology, National Cancer Centre Singapore, Singapore; d Division of Medical Sciences, National Cancer Centre Singapore, Singapore; e Oncology Academic Programme, Duke-NUS Medical School, Singapore; f Department of Radiation Oncology, Northern Sydney Cancer Centre, Royal North Shore Hospital, Sydney, New South Wales, Australia; g Department of Radiation Oncology and Image-Applied Therapy, Graduate School of Medicine, Kyoto University, Kyoto, Japan; h Department of Radiation Oncology, St George Hospital, Sydney, New South Wales, Australia; i Department of Radiology, University of Yamanashi, Japan; j Department of Radiation Oncology, Asan Medical Center, University of Ulsan College of Medicine, Seoul, Republic of Korea; k Department of Radiation Oncology, American University of Beirut Medical Center, Beirut, Lebanon; l Baptist Health, Lynn Cancer Institute, Boca Raton, Florida; m Varian Medical Systems, Palo Alto, California

## Abstract

Hypofractionated radiotherapy schedules provide higher per-fraction radiation doses delivered in fewer fractions than conventional schedules. This novel delivery method is supported by a large body of clinical trial evidence across various cancer sites in both curative and palliative settings. Hypofractionation is associated with benefits such as lower costs, improved patient access and increased treatment precision, which has led to its inclusion in various treatment guidelines. Despite this, utilization is not uniform across cancer sites and geographic regions due to reasons such as reimbursement models, nuances in healthcare systems, and professional culture. Key factors to ensure patients benefit from access to high quality radiotherapy include publishing clinical evidence, cross-country collaboration to fill knowledge gaps, reviewing reimbursement models, and improving patient advocacy in treatment decision-making.

## Introduction

Hypofractionated radiation therapy schedules, which provide higher per-fraction radiation doses delivered in fewer fractions than conventional schedules, are supported by a large body of clinical trial evidence across various cancer sites in both curative and palliative settings.^1^, ^2^, ^3^, ^4^ These data demonstrate at least noninferior tumor control without increasing severe toxicity compared with conventional methods. Such studies have subsequently supported the inclusion of hypofractionation in treatment guidelines as a suitable regimen for various cancers, including those of the brain, breast, lung, and prostate.^5^, ^6^, ^7^, ^8^, ^9^ Hypofractionation can also lead to lower costs and improved patient access relative to conventional schedules.^10^

Nevertheless, it has been noted that hypofractionation utilization is not uniform among cancer sites or geographic regions. In particular, there is a considerable lag in adoption in the Asia-Pacific region compared with North America and Europe.^10^ These may stem from nuances in local healthcare systems, reimbursement models, and professional culture, among other reasons, which ultimately impede the delivery of affordable and accessible radiation therapy.

This article describes the expert opinions and personal experiences of radiation oncologists from South Korea, Japan, Singapore, and Australia to represent a snapshot of the Asia-Pacific region, with the goal of highlighting specific tumor sites where emerging issues regarding practice patterns and barriers to utilization exist within our respective countries. We focus on tumors of the brain, breast, lung, and prostate to give an informative account of where variations in practice occur; however, it is important to note that there are other common tumors in the Asia-Pacific region not covered in this article, and more research and information is needed surrounding these tumors to add to the growing conversation around the role of hypofractionation in the region. Based on our observations, we provide possible solutions to optimize the use of hypofractionation for these select tumor sites that may ultimately help to improve patient access in Asia-Pacific countries.

## Current Utilization of Hypofractionation

Conventional fractionation schedules, although effective, can entail 5 to 8 weeks of radiation therapy delivered in 1.8 Gy to 2.0 Gy fractions, which impose considerable time and financial burdens on patients. Hypofractionation (ie, doses ranging from >2.0 Gy per fraction for moderate hypofractionation to ≥5.0 Gy for ultrahypofractionation^10^^,^^11^) means fewer fractions are needed, thereby reducing overall treatment time and cost while liberating machine time and improving access for a greater number of patients. This has been of particular benefit throughout the recent COVID-19 pandemic, where shorter treatment courses reduced patient-hospital exposure and mitigated the risk of infection and interruption of radiation therapy.^10^ Advanced intensity modulation and image guidance technologies allow for increased precision and steeper radiation fall-off, thus leading to less exposure of surrounding normal tissues to toxic levels of radiation. These technological advances make the use of ultrahypofractionated methods, such as stereotactic radiosurgery and stereotactic body radiation therapy (SBRT), safe for tumors throughout the body.^11^

Despite these clinical advantages, hypofractionation utilization is lacking in specific countries of the Asia-Pacific region. A recent global study has highlighted disparities by geographic region and cancer site, including breast and prostate.^10^ Out of 2316 radiation oncologists included in the study, it was found that hypofractionation was preferred by 82.2% of respondents for node-negative breast cancer after lumpectomy, with the highest proportion of hypofractionation users in Europe (88.5%) and North America (97.3%; *P* < .001), whereas respondents in the Asia-Pacific region were among the lowest proportion of users (72.0%). Overall preference for hypofractionation was significantly reduced in the postmastectomy setting, with the lowest rates found in Asia-Pacific (36.2%; *P* = .002). Similar findings were observed for node-positive disease.^10^ Amongst respondents, low- and intermediate-risk prostate cancer saw high total hypofractionation utilization, with the highest rates again being seen in North America (94.3% low risk, 87.8% intermediate risk; *P* < .001). In comparison, the Asia-Pacific region appeared to have average utilization at this site (42.0% low risk, 41.0% intermediate risk). Compared with curative indications, high utilization rates of hypofractionation for palliation in both breast and prostate cancer were reported in all geographies.^10^

Although these data cannot be extrapolated to the brain and lung due to nuances in treatment, we see a clear trend among radiation oncologists in North America and Europe who have proven to be early adopters of hypofractionation and so may reap its advantages. In contrast, their Asian-Pacific counterparts appear to be lagging behind,^10^ raising the question, “Why?”

From our experience in Australia, moderate hypofractionation for breast cancer has become the standard of care, due in part to the solid base of high-level data available within early breast cancer coupled with its significant health and economic benefits. In practice, hypofractionation tends to be more frequently used in older women, as some physicians have concerns about the late toxicity of hypofractionation, adjuvant chemotherapy, and effects on younger patients postmastectomy. We also note that hypofractionation is well suited to the prostate, and dose escalation has been proven to achieve optimal control in intermediate- to high-risk cases, leading to guideline inclusion. Prostate SBRT is now the standard of care in Singapore, in our opinion, and this has facilitated the development of reimbursement schemes for moderate hypofractionation. In contrast, prostate hypofractionation utilization is low in Japan; nonetheless, there has been a steady increase in utilization here throughout the COVID-19 pandemic, possibly due to the shifts in clinical practices accelerated by the pandemic in addition to reimbursement by the national health insurance initiated in 2018.

For brain malignancies, our experience in Australia is that moderate hypofractionation is usually reserved for patients ≥75 years old with an Eastern Cooperative Oncology Group performance status of ≥2 for glioma. These observations are mirrored in published data from North America.^12^ It has been observed that utilization rates for hypofractionation are usually higher in academic facilities and metropolitan areas, and hypofractionated schedules are more commonly used for patients living >50 miles away from their treatment center. For lung malignancies, in our experience, SBRT is commonly used to treat metastatic tumors in South Korea as it is widely recognized as effective. In contrast, high-dose radiation therapy utilization for primary tumors is low in Japan, and surgery is the standard of care due to the proximity of the lung to vital organs and structures. Although SBRT is considered appropriate for those with high surgical risk (eg, >75 years old or poor lung function), there is no age limit for surgery in Japan, so surgeons often prefer this route to gain tumor pathology and mutation/receptor status.

## Barriers to Adoption of Hypofractionation

Several barriers to hypofractionation utilization and patient access have been reported in the literature and are echoed by our experience. Reimbursement is a key driver that heavily influences the adoption of hypofractionation, and its effect seems more pronounced in the Asia-Pacific region,^10^ where we observe lower remuneration for hypofractionation than conventional schedules. Pay-per-fraction models in countries such as Japan disincentivize physicians to choose shorter treatment schedules and remove the physicians’ flexibility to choose the optimal treatment platform and the number of fractions best suited to the patient. In Singapore, treatment claims are assessed on the number of fractions and mode of delivery (eg, conventional image-guided versus SBRT methods). SBRT is subjected to a higher claim quantum per fraction to offset the net cost of the advanced technique, which otherwise would cost substantially more than a longer course of moderately hypofractionated radiation therapy. In South Korea, 1 to 4 fractions are considered SBRT; however, there are no specialized payment systems for >4 fractions.

Although most radiation therapies are covered by national healthcare insurance, in some countries, we observe that differences between public and private sector payment methods can also pose challenges for patients. For instance, in Australia, although the federal government funds all radiation oncology services provided in the public sector under the national healthcare insurance scheme, out-of-pocket costs above insurance coverage for services provided in the private sector are charged to the patient on a case-by-case basis. Radiation therapy is mostly based on a fee-for-service system in South Korea and Japan, and both national health insurance systems require copayments from patients but have a cap to protect against excessive costs. Contrary to South Korea, Japan permits additional fees for image-guided radiation therapy, tumor motion tracking, and hypofractionation. Only locally-based solid malignant tumors are reimbursed for the expense of image-guided radiation therapy in Japan, whereas metastatic lesions are also covered in South Korea.^13^

Despite its growing evidence base and its inclusion in treatment guidelines, concerns around toxicity and late effects of hypofractionation are a significant barrier, especially for certain anatomic sites.^10^ We agree that knowledge gaps certainly exist for breast cancer, including postmastectomy, nodal treatment, and treatment of younger patients. Most primary brain tumor data are on glioma in elderly patients, so additional randomized controlled trials are needed within the remit of anaplastic and other central nervous system tumors in younger patients. Likewise, more data are required for ultrahypofractionation and SBRT for the prostate, and comparability between surgery and SBRT may facilitate greater adoption of lung hypofractionation.

We believe that certain practice cultures can hinder adoption, as there are many pressures on physicians around patient advocacy and early adoption of novel techniques, which is particularly heightened in the Asia-Pacific region. Additionally, the opinions of surgeons and medical oncologists pose a significant barrier to radiation therapy and the referral pathway, and it is felt that radiation oncologists’ opinions are sometimes overruled within the multidisciplinary team. As surgeons are responsible for diagnostic biopsies at most sites, they are the gatekeepers to treatment. However, in our experience, surgeons often present limited information to patients about radiation therapy and do not always refer patients to radiation oncology specialists, indicating discrepancies in hospital policies and referral pathways.^14^^,^^15^

Training, administrative abilities, and local expertise also affect utilization, and the lack of availability of technology and radiation machines, especially in rural clinics, exacerbates the problem. In our experience in Singapore, the evolving oncology landscape and the emergence of new treatment modalities have further contributed to a decrease in referrals for hypofractionation, notably for brain metastases.

## Solutions to Optimize Hypofractionation Utilization

These challenges highlight the need to identify solutions that optimize the use of hypofractionation schedules to improve radiation therapy availability, accessibility, and affordability for patients (Table 1). The most significant opportunity lies within provider-reimbursement models, as they appear to have the biggest disparity between anatomic sites and geographic regions. It may be beneficial to make information regarding national and international hypofractionation reimbursement models (including details on reimbursement per fraction and total cost) publicly available to help physicians compare with global peers and gain fair compensation. We have seen that bundled payment for care improvement schemes in the United States may provide a total cost for care regardless of fraction number, thus potentially putting emphasis on rewarding the quality of care provided rather than the quantity of service offered by providers. Reworking remuneration models to account for patient outcomes, advanced techniques, adherence to evidence-based practice while considering feedback from health economics and outcomes research, and facilitating communication between government agencies, providers, and payers may allow countries in the Asia-Pacific region to move away from pay-per-fraction models and incentivize hypofractionation adoption.

**Table 1:** Identified barriers and solutions to optimize patient access to hypofractionated radiation therapy in cancer care based on observations from the Asia-Pacific region at select cancer sites

Barrier	Solution
Inadequate provider reimbursement, especially when considering advanced techniques	Reworking provider remuneration models to move away from pay-per-fraction models and account for patient outcomes, use of advanced techniques, and adherence to evidence-based practice Making national and international hypofractionation reimbursement models publicly available Facilitating communication between government agencies, providers, and payers
Lack of available resources, administrative abilities, or technology	Applying lessons from COVID-19 effect (eg, appointment scheduling) to radiation therapy
Barriers within professional culture and ununified multidisciplinary teams	Increased clinical trial data to ease concerns regarding radiation toxicity Promoting a multidisciplinary approach to treatment planning
Gaps in the clinical pathway, including a lack of referrals and an absence of joint decision-making	Patient advocacy Patient/physician education Joint decision-making with patients
Knowledge gaps and clinical evidence needs (eg, clinical trial data, real-world evidence, cost-effectiveness data)	Increased data generation to fill gaps and bolster confidence
The evolving oncology landscape (eg, new methods of investigation and treatment)	Increased research and development efforts within hypofractionation

Another opportunity lies in reinforcing referral schemes by linking them to remuneration. Recently, the Australian reimbursement scheme was updated to state that, for urologists to receive full remuneration, all patients with prostate cancer must be encouraged to discuss treatment options with a radiation oncologist.^16^ This best-practice motion could be applied to other countries and sites; standardizing referral pathways, heightening patient advocacy, and ensuring that patients see a radiation oncologist have the potential to ensure that patients receive accurate information about their treatment options and reduce variability in practice patterns. Other solutions to optimize hypofractionation utilization include increased awareness of clinical evidence throughout cancer sites among surgeons and medical oncologists to fill knowledge gaps, ease concerns, and gain buy-in, in addition to developing evidence-based clinical guidelines for various cancer sites. Also, applying lessons from the effect of COVID-19 may help to ease a lack of resources, whereas increased research and development potentials within hypofractionation may help to keep the treatment at the forefront.

## Conclusion

Hypofractionation is indeed an important yet underutilized radiation therapy technique, and we encourage the appropriate use of hypofractionation when supported by high-level evidence and the individual patient's clinical presentation. Despite general progress in adoption, there is significant variability across geographic regions and disease sites, and our observations highlight the need for targeted interventions that address specific barriers to hypofractionation and support evidence-based adoption. Regarding future directions, we believe that increased clinical evidence could help to fill knowledge gaps and promote confidence in treatment. One way to achieve this is by combining data gathered from collaborative research studies and real-world evidence to create sizeable databases for appropriate outcome analysis. There is also a need for cross-country collaboration to facilitate education, where experts in countries with significant hypofractionation experience showcase their best practices to physicians in countries with less experience. Centers of excellence can also be established for experts to further share knowledge and conduct preceptorships. Reimbursement is indeed an important factor for utilization, and overhauled models that ensure fair compensation within the Asia-Pacific region may help in this regard. Lastly, it is critical to uphold the role of radiation oncologists within the multidisciplinary team in advocating for patients, educating peers on data, and facilitating effective treatment decision-making to ensure patients benefit from this significant advancement in modern radiation therapy.

## Disclosures

Melvin L.K. Chua reports consulting fees from ImmunoSCAPE and Telix Pharmaceuticals; honoraria from Astellas, Bayer, MSD, Janssen, Pfizer, and Varian Medical Systems; personal fees from AstraZeneca and Ferring; and nonfinancial support from AstraZeneca, Decipher Biosciences, and MedLever. He also has stocks in Digital Life Line, is a co-inventor and co-owner of the patent of a high sensitivity lateral flow immunoassay for detection of analyte in sample (10202107837T), Singapore, and is supported by the National Medical Research Council Singapore Clinician Scientist Award (NMRC/CSA-INV/0027/2018, CSAINV20nov-0021), the Duke-NUS Oncology Academic Program Goh Foundation Proton Research Programme, NCCS Cancer Fund, and the Kua Hong Pak Head and Neck Cancer Research Programme. Dasantha Jayamanne has received honoraria from Varian Medical Systems. Takashi Mizowaki has received grants from Varian Medical Systems, Hitachi Ltd, and Brainlab AG; consulting fees from Varian Medical Systems; and honoraria from Varian Medical Systems, Hitachi Ltd, Elekta, and Brainlab AG. Si Yeol Song has received honoraria from IQVIA. RAS is an employee of Varian Medical Systems, a Siemens Healthineers company.
